# Prof. Chapin A. Harris

**Published:** 1893-05

**Authors:** 


### PROF. CHAPIN A. HARRIS.

It is sometimes pleasant to have a peep of the inner life of our great men. We have just received two or three private letters written by Prof. C. A. Harris, one of the original promoters of the Baltimore Dental Colleges, and author of our best medico-dental dictionaries. They were written to his brother, Thomas W. Harris, of Littleton, N. C. They reveal a warm heart, a genial nature, and a sociality that we do not always find in our prominent public men.

In a letter written March 12th, 1839, among other things, he reveals the fact that he had been an inveterate user of tobacco; but, while on a visit to his brother, had been persuaded, with others, to give it up. This resulted in the formation there of an "Anti-Using Tobacco Society." It also shows that since returning to Baltimore he had been sorely tempted to backslide. What a terrible strong habit it must be! He says:

"How does the Anti-Using Tobacco Society come on? Who would have thought the crop would have been so great? Would you not like to join me in a social smoke? How vastly pleasant it would be to spend an evening as we were wont to do while I was at your house? Ah, I often think about it, and have longed to do it again, but cannot say I have shed many 'hogsheads of tears,' though I can assure you its deprivation has cost me many a sorrowful hour. At times it has caused me to wish I had never joined the society. To be plain, have you not some notion of withdrawing from the society? I must confess I have, but this I do wish you not to mention. I work for the society, however, and am trying to hold out. After I left your house I obtained the following names which I here beg leave to put on the records of the society."

As it must be a pleasure to the many descendants of these persons still living to see this list, we transcribe them.

"Dear Madam:

"The following persons have authorized me to put their names down as members of the N. C. Anti-Using Tobacco Society. Rev. Wm. Burge, Mrs. Wm. Burge, Miss Rebecca Ann Burge, Miss Mary Jane Burge, James P. Burge, Miss Avy Robert, Mr. Henry Harris, Mrs. Henry Harris, Miss Sarah Clanton, Mrs. Edward Alston, Miss Martha Crondup, Mrs. H. H. Williams, Miss Ann Brickle, Mrs. Mary R. Alston, Hon. Wm. Branch, Mrs. Wm. Branch, Miss Sarah Bennens."

It may be thought singular that so many ladies joined this society. But we understand that part of the pledge was that the ladies should not accept the company of gentlemen who used tobacco, and that even married ladies should do their best to discourage its use by their husbands and children. Also, it must be remembered, that then many girls and women smoked, and rubbed finely ground tobacco on their teeth. I have seen a few such in the South in my day; but, of course, not of the respectable class. How revolting it does look in a woman? And should not the using of tobacco look quite as bad in a man?

Dr. Harris was a Methodist, and quite active in promoting the more zealous and spiritual interests of Christianity.—*Items of Interest.*

---

